# Natural Nrf2 Modulators for Skin Protection

**DOI:** 10.3390/antiox9090812

**Published:** 2020-09-01

**Authors:** Yong Chool Boo

**Affiliations:** Department of Molecular Medicine, School of Medicine, BK21 Plus KNU Biomedical Convergence Program, Cell and Matrix Research Institute, Kyungpook National University, Daegu 41944, Korea; ycboo@knu.ac.kr; Tel.: +82-53-420-4946

**Keywords:** nuclear factor erythroid 2-related factor 2 (Nrf2), antioxidant responsive elements (ARE), kelch-like ECH-associated protein 1 (Keap1), β-transducin repeat-containing protein (β-TrCP), phosphorylation, acetylation, natural products, terrestrial and marine plants, inflammation, aging, cancer

## Abstract

Since the discovery of antioxidant responsive elements (ARE), which are commonly found in the promoter of the Phase II metabolism/antioxidant enzymes, and nuclear factor erythroid 2-related factor 2 (Nrf2), the transcription factor that binds to ARE, the study conducted in this field has expanded remarkably over the decades, and the Nrf2-mediated pathway is now recognized to occupy a central position in cell defense mechanisms. Induction of the Phase II metabolism/antioxidant enzymes through direct activation of Nrf2 can be a promising strategy for preventing degenerative diseases in general, but a dark side of this strategy should be considered, as Nrf2 activation can enhance the survival of cancer cells. In this review, we discuss the historical discovery of Nrf2 and the regulatory mechanism of the Nrf2-mediated pathway, focusing on the interacting proteins and post-translational modifications. In addition, we discuss the latest studies that examined various natural Nrf2 modulators for the protective roles in the skin, in consideration of their dermatological and cosmetic applications. Studies are reviewed in the order of time of research as much as possible, to help understand how and why such studies were conducted under the circumstances of that time. We hope that this review can serve as a steppingstone in conducting more advanced research by providing a scientific basis for researchers newly entering this field.

## 1. Introduction

Because the skin is a body organ that is directly exposed to the external environment, a special defense strategy is needed for attacks by ultraviolet (UV) rays, contaminants, physical wounds, and biological hazards [1,2,3,4]. Skin protection is basically dependent on the physical and chemical barrier function of the stratum corneum in the skin [5]. However, failure of these functions can lead to serious skin diseases and premature skin aging [6]. Even if these functions approximately occur, it is not possible to completely avoid skin cancer and many other diseases. Therefore, research on artificial auxiliary means for prevention and treatment of skin diseases is of great importance and need [7].

Skin damage caused by internal and external factors is accompanied by oxidative stress in many cases [8,9]. Thus, antioxidants that can directly scavenge the free radicals, and alleviate the inflammatory response mediated by the free radicals are expected to provide a beneficial effect in the skin [3,4]. However, before the auxiliary means are provided externally, the skin cells are prepared with various forms of defense against oxidative stress, and among them, nuclear factor erythroid 2-related factor 2 (Nrf2)-mediated pathway is recognized to be the most important one [10].

Cells use a strategy to enhance their own defense capabilities prior to receiving severe damage that cannot be safely addressed, by sensing the oxidative insults as early as possible, and by promoting the expression of Phase 2 metabolism/antioxidant enzymes, such as glutathione S-transferase (GST), nicotinamide adenine dinucleotide (phosphate) (NAD(P)H) quinone oxidoreductase-1 (NQO-1), heme oxygenase-1 (HO-1), γ-glutamate-cysteine ligase catalytic subunit (GCLC) and its regulatory subunit (GCLM), glutathione peroxidase (GPX), superoxide dismutase (SOD), uridine diphosphate (UDP)-glucuronosyltransferase 1A1 (UGT1A1), 8-oxoguanine DNA glycosylase 1 (OGG1) [11,12]. Cells use a strategy to alter their surrounding microenvironment to a more reductive state to be prepared for the expected oxidative stress [13]. The factors that cause oxidative damage and inflammatory response of the cells are diverse, and the process of detecting and transmitting the change, and the process of waking up the defense system are also very complicated. One of the important goals of this review is to help readers understand the basics of the Nrf2-mediated pathway.

Another goal of this review is to discuss recent studies aiming at enhancing the defense of skin cells using natural substances that modulate the Nrf2-mediated pathway. We hope that this review will serve as an orientation for researchers newly entering this field. Historically important studies were described in chronological order as much as possible to make it easier to understand the situation at the time of the study. Additionally, our subjective judgment did not assess the importance of the findings, and we did more to introduce them from an objective perspective.

Would it not be better to prevent a variety of diseases if we could operate the cell’s own defense system with a small amount of a Nrf2 modulator rather than providing a large amount of an antioxidant to reach enough concentrations to remove the free radicals inside the cells? Let us try to find a right answer!

## 2. The Nrf2-Mediated Pathway

### 2.1. Discovery the Nrf2/ARE-Mediated Cell Defense Mechanism

In 1988–1989, Telakowski-Hopkins et al. and Daniel et al. reported that the 5′-flanking region of a rat or mouse *GST Ya subunit* gene contains two cis-acting regulatory elements, one of which is required for constitutive expression, and the other is required for inducible expression in response to planar aromatic compounds, such as β-naphthoflavone [14,15]. The cis-acting element required for inducible expression was responsive to planar aromatic compounds only in cells with a functional aryl hydrocarbon receptors (AhR) and cytochrome P1-450 activity, whereas it directly responded to electrophilic inducers such as trans-4-phenyl-3-buten-2-one, dimethyl fumarate, and t-butylhydroquinone, thus the element was designated as electrophile-responsive elements (EpRE) by Friling et al. in 1990 [16].

In 1990, Rushmore et al. identified a xenobiotic-responsive element in the 5′ flanking sequence of a *GST Ya subunit* gene partly responsible for the basal level as well as inducible expression of the Ya subunit gene by planar aromatic compounds, such as β-naphthoflavone [17]. The element was directly responsive to phenolic antioxidants, such as t-butylhydroquinone, and it was named antioxidant-responsive elements (ARE) [18]. Mutational and deletion analyses of the *GST Ya subunit* gene and the *NQO-1* gene identified the core sequence of the ARE; 5′-puGTGACNNNGC-3′, 3′-pyCACTGNNNCG-5′, where N is any nucleotide [19]. ARE and EpRE appeared to be the identical deoxyribonucleic acid (DNA) sequences similar to activator protein-1 (AP-1) binding sites [20].

A tandem-repeated consensus sequence is present in the β-globin locus control region and binds to the AP-1 family and/or to the nuclear factor erythroid 2 (NF-E2) [21]. In 1993, Dr. Kan’s research team screened a human erythroleukemia cell line (K562) complementary DNA (cDNA) library using the tandem repeat as a recognition site probe, and isolated several cDNAs, two of which had remarkable similarities with the genes encoding NF-E2, thus the novel gene products were named Nrf1 and Nrf2 [22,23]. *Nrf1* and *Nrf2* genes encode members of the basic leucine zipper (b-zip) protein family that activate transcription via the DNA binding domain highly homologous to that of NF-E2. A high degree of homology is found in the b-zip and neighboring regions among Nrf2, Nrf1, NF-E2, and the Drosophila segmentation protein Cap ‘N’ Collar (CNC) [22,23]. Nrf1 and Nrf2 are different in molecular size and overall structure but share several homology domains [24].

Chan et al. reported that Nrf2 was not essential for growth, development, or erythropoiesis in mammalian cells based on the observation in *Nrf2* knockout mice [25], but a role of Nrf2 in the regulation of hematopoietic stem cell function was substantiated later by Tsai et al. [26].

### 2.2. Keap1-Dependent Regulation of Nrf2

After AREs had been known to be involved in the transcriptional control of a group of phase II detoxifying enzymes, the identity of Nrf2 as a transcription factor that acts through AREs was revealed by Itoh et al. using homozygous *Nrf2*-mutant mice [10]. Unlike *Nrf2* knockout mice, *Nrf1* knockout mice displayed anemia due to impaired fetal liver erythropoiesis and embryonic lethality [27]. Nrf1 upregulated a unique battery of ARE-dependent genes, such as *metallothionein (MT)-1* and *-2* in addition to typical Nrf2 target genes, such as *NQO-1* [28]. Nrf3 was also recognized in 1999 [29]. *Nrf3* knockout mice displayed no overt phenotype [30]. All Nrf members, such as Nrf1, -2, and -3, regulate the expression of ARE-dependent genes, with differential and overlapping DNA-binding and transcriptional activities, and they show differed tissue distribution [31].

In 1999, Itoh et al. observed that the deletion of Nrf2-ECH (erythroid cell-derived protein with CNC homology) homology (Neh) 2 domain of Nrf2 led to more potent transactivation in erythroblasts, indicating that Nrf2 activity is normally repressed and the Neh2 domain is involved in the negative regulation. Using the yeast two-hybrid system, they discovered a novel cytoplasmic protein that binds to Nrf2 through the Neh2 domain, and negatively regulates the transactivation potential of Nrf2, which was named Kelch-like ECH-associated protein 1 (Keap1) [32] because of its structural similarity to a Drosophila actin binding protein called Kelch [33] and chicken ECH [34]. They further found that Nrf2 bound to Keap1 is rapidly degraded through the proteasome pathway, while electrophiles can liberate Nrf2 from Keap1, and cause Nrf2 nuclear translocation with concomitant stabilization [35].

In 2002, Zipper et al. showed that the two Keap1 molecules form a homodimer via their Broad-Complex, Tramtrack and Bric-a-Brac (BTB) domains, and Keap1 dimerization is required for Nrf2 sequestration and transcriptional repression [36]. Dinkova-Kostova et al. proposed that the modification of sulfhydryl groups of certain cysteine (Cys, C) residues in Keap1, rather than Nrf2, may be important to the regulation of the Nrf2 pathway, as the Neh2 region of Nrf2 contains no Cys residues [37]. A number of Cys residues in the intervening region (IVR) between the BTB domain and Kelch-repeats of the Keap1 protein, such as Cys257, Cys273, Cys288, and Cys297, were found to be particularly reactive [37].

In 2003, Zhang et al. identified redox-sensitive Cys residues in Keap1, including Cys151, that were required for Keap1-dependent ubiquitylation of Nrf2 and degradation by proteasomes [38]. They proposed that Keap1 is a redox-regulated substrate adaptor protein for a cullin (Cul) 3-dependent E3 ubiquitin ligase complex that is specifically targeted for inhibition by oxidative stress [39].

The Neh2 domain of Nrf2 has both ^29^DLG^31^ and ^79^ETGE^82^ motifs [40,41], one of which motifs binds to one molecule of Keap1, and the other binds to a second molecule of Keap1 [42], bringing Nrf2 to the proximity of Cul3, a scaffold protein that forms the E3 ligase complex with really interesting new gene (RING)-box 1 (RBX1) [43,44,45]. Under the basal, unstressed condition, the Cul3 complex ubiquitylates Nrf2 at a lysine (Lys, K) residing between the ^29^DLG^31^ and ^79^ETGE^82^ motifs [42].

In 2005, Velichkova et al. showed that Keap1 sequestered Nrf2 in the cytoplasm through an active chromosome region maintenance 1 (Crm1)/exportin-1-dependent nuclear export mechanism [46]. They identified a nuclear export signal (NES) consensus sequence in the IVR of Keap1, and mutation of hydrophobic amino acids in the NES sequence resulted in nuclear accumulation of Keap1 and Nrf2, as did leptomycin B, which inactivates Crm1. Thus, a modification of Keap1’s NES was proposed to promote the entry of both Keap1 and Nrf2 into the nucleus and transcriptional transactivation of ARE-driven genes.

**Figure 1:**
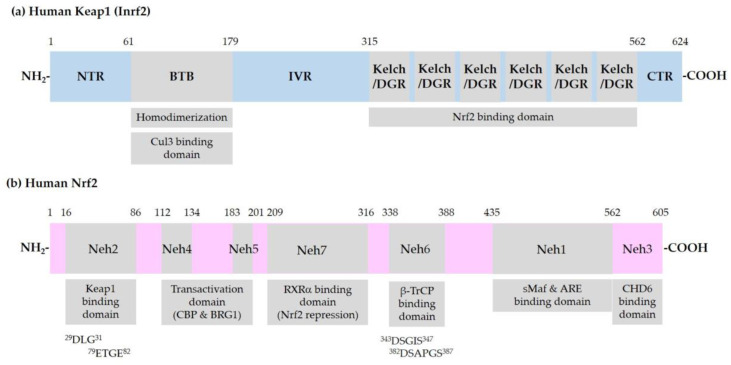
Schematic representation of domain structures of Kelch-like ECH-associated protein 1 (Keap1) and nuclear factor erythroid 2-related factor 2 (Nrf2). (**a**) Functional domains of Keap1: N-terminal region (NTR), Broad-Complex, Tramtrack, and Bric-a-Brac (BTB) domain, intervening region (IVR), Kelch/Double glycine repeats (DGR) domain, and C-terminal region (CTR). BTB is responsible for homodimerization between two Keap1 molecules and association with a Cullin (Cul) 3/E3 ubiquitin ligase complex. IVR contains reactive cysteine (Cys) residues. Kelch/DGR domain is responsible for binding to ^29^DLG^31^ and ^79^ETGE^82^ motifs in the Nrf2-ECH homology (Neh) 2 domain of Nrf2; (**b**) Neh domains of Nrf2: Neh1 domain is the binding site for small musculoaponeurotic fibrosarcoma (sMaf) proteins and antioxidant response element (ARE) in the promoter region of deoxyribonucleic acid (DNA). Neh2 domain contains ^29^DLG^31^ and ^79^ETGE^82^ motifs, through which Nrf2 interacts with Kelch/DGR domains of two Keap1 molecules of a homodimer. Neh6 domain contains ^343^DSGIS^347^ and ^382^DSAPGS^387^ motifs, through which Nrf2 interacts with β-transducin repeat-containing protein (β-TrCP). Neh4 and -5 domains are involved in transcriptional activation. Binding domain for cAMP-responsive element binding protein (CREB) binding protein (CBP), Brahma-related gene 1 (BRG1), chromo-ATPase/helicase DNA binding protein (CHD6), and retinoic X receptor α (RXRα) are indicated.

Figure 1 shows the domain structures of Keap1 and Nrf2.

**Figure 2:**
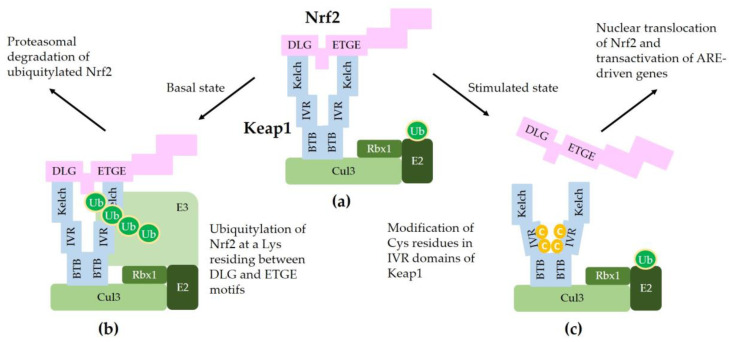
A model for interaction between Nrf2 and Keap1 in the canonical Nrf2 pathway. (**a**) Two molecules of Keap1 form a homodimer through the BTB domain and bind to Cul3. Dimeric keap1 molecules capture a Nrf2 molecule through the Kelch/DGR domains that interact with the ^29^DLG^31^ and ^79^ETGE^82^ motifs of Nrf2, resulting in the sequestration of Nrf2 to a Cul3/really interesting new gene (RING)-box 1 (RBX1)/E3 ubiquitin ligase complex. (**b**) Under basal, unstressed conditions, the Cul3 complex ubiquitylates Nrf2 at a lysine (Lys, K) residue between the ^29^DLG^31^ and ^79^ETGE^82^ motifs. Ubiquitylated Nrf2 is then transferred to the 26S proteasomes, where it is degraded. (**c**) When cells are stimulated by oxidants or xenobiotics, certain cysteine (Cys, C) residues of Keap1 can be modified, thereby preventing ubiquitylation and releasing Nrf2 protein. The released Nrf2 can translocate to the nucleus and get involved in the transcriptional activation of ARE resulting in the enhanced gene expression of target genes.

Figure 2 illustrates a schematic model for sequestration of Nrf2 by a Keap1/Cul3/Rbx1/E3 ubiquitin ligase complex and subsequent ubiquitylation for proteasomal degradation of Nrf2.

### 2.3. Other Proteins that Interact with Nrf2 or ARE

In 1994, Igarashi et al. showed that the small musculoaponeurotic fibrosarcoma (sMaf) proteins, which possess a b-zip DNA binding domain but lack a canonical transactivation domain, directly associate with Nrf2 that also contains a b-zip domain, conferring DNA binding property. Heterodimers of sMaf and Nrf2 induced active transcription whereas homodimers of sMaf molecules act as negative regulators, indicating that positive and negative regulation of transcription can be achieved by controlling the relative concentrations of Nrf2 and sMaf in the nucleus [47].

In 2001, Katoh et al. found that two transcription activation domains of Nrf2, Neh4 and Neh5, cooperatively bind to a transcriptional coactivator, cyclic AMP-responsive element binding protein (CREB) binding protein (CBP) [48]. In 2007, Zhang et al. showed that binding of CBP and Brahma-related gene 1 (BRG1) to Neh5 domain of Nrf2 enhanced HO-1 promoter activity cooperatively [49].

In 2005, Dhakshinamoorthy et al. demonstrated that BTB domain and CNC homolog 1 (Bach1) binds to ARE as a heterodimer with sMaf proteins but not as a homodimer or heterodimer with Nrf2, and thereby competes with Nrf2 leading to the suppression of ARE-mediated *NQO-1* gene expression [50]. Nioi et al. found that the Neh3 domain of Nrf2 is needed for its interaction with chromo-ATPase/helicase DNA binding protein 6 (CHD6) to induce transcriptional activity, regulating *NQO-1* gene expression [51]

In 2013, Wang et al. reported a function of retinoic X receptor α (RXRα) as a Nrf2 repressor. They observed that RXRα physically interacts with Nrf2 and binds to ARE sequences in the promoters of Nrf2-regulated genes, and suggested a hypothesis that a direct interaction between Nrf2 and RXRα on gene promoters accounts for the antagonism of ARE-driven gene expression. They designated the Neh7 domain, comprising amino acids 209–316 in human Nrf2, for interaction with the DNA binding domain of RXRα.

In 2012, Rojo et al. observed that a cancer-chemopreventive agent, nordihydroguaiaretic acid (NDGA), increased the level of Nrf2 protein and expression of HO-1 in wild-type mouse embryo fibroblasts (MEFs) and in *Keap1(-/-)* MEFs, but not in *Nrf2(-/-)* MEFs, implying that Keap1-independent mechanisms regulate Nrf2 stability and HO-1 induction [52]. NDGA caused inhibitory phosphorylation of glycogen synthase kinase 3β (GSK3β), and this was associated with a reduction in Neh6 phosphorylation. Subsequently, two serine (Ser, S) residues, Ser344 and Ser347 (Mouse Ser335 and Ser338) in the Neh6 domain of Nrf2, were identified to be phosphorylated by GSK3 [53].

In 2013, Chowdhry et al. proved that Nrf2 can be ubiquitylated by a β-transducin repeat-containing protein (β-TrCP)/S-Phase kinase-associated protein (Skp) 1/Cul1/Rbx1/E3 ubiquitin ligase complex [54]. In this complex, β-TrCP acts as a substrate receptor and captures Nrf2 through binding to ^343^DSGIS^347^ and ^382^DSAPGS^387^ motifs that are present in the Neh6 domain of Nrf2. GSK3-mediated phosphorylation of Ser residues in the ^343^DSGIS^347^ motif enhanced the interaction between Nrf2 and β-TrCP, which resulted in increased ubiquitylation of Nrf2 and subsequent proteasomal degradation. As the function of β-TrCP in the regulation of Nrf2 stability is distinct from Keap2, Dr. Cuadrado has proposed “Dual nuclear flux control model for regulation of Nrf2 stability by Keap1” by defining the roles of Keap1and β-TrCP as a “limiter valve” and a “regulator valve,” respectively, to control the nuclear flux of Nrf2 [55].

There are many other proteins that are involved in the regulation of the Nrf2 pathway, such as the endoplasmic reticulum (ER)-associated E3 ubiquitin ligase, 3-hydroxy-3-methylglutaryl-coenzyme A (HMG-CoA) reductase degradation 1 (HRD1), that interacts with Nrf2 through Neh4/5 domains and promotes the proteasomal degradation of Nrf2 [56], and the scaffold protein Caveolin 1 that was proposed to compete with Keap1 for Nrf2 binding, thereby enhancing the stability of Nrf2 [57], but all cannot be covered here due to my limited capability.

**Figure 3:**
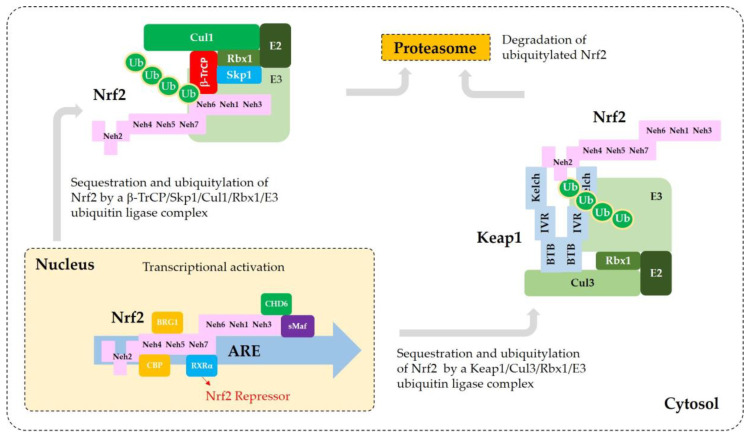
A model for the interaction of Nrf2 with various proteins that promote sequestration of Nrf2 or enhance or suppress its transcriptional activity. In the nucleus, heterodimerization of Nrf2 with sMaf through its Neh1 domain is required for efficient binding to the ARE in the promoter region of DNA. Binding of CBP and BRG1 at Neh4/5 domains and CHD6 at Neh3 domain of Nrf2 are required for full transcriptional activity of Nrf2. RXRα binds to Neh7 domain of Nrf2, acting as a repressor. In the cytosol, Keap1 captures Nrf2 through binding to ^29^DLG^31^ and ^79^ETGE^82^ motifs in Neh2 domain and sequesters it to a Cul3/Rbx1/E3 ubiquitin ligase complex. Alternatively, β-TrCP captures Nrf2 through binding to ^343^DSGIS^347^ and ^382^DSAPGS^387^ motifs in Neh6 domain and sequesters it to a Cul1/Skp1/Rbx1/E3 ubiquitin ligase complex. Ubiquitylated Nrf2 is then transferred to the 26S proteasomes, where it is degraded.

Figure 3 illustrates the interaction of Nrf2 with various proteins that are known to regulate the sequestration of Nrf2 or its transcriptional activity.

### 2.4. Phosphorylation of Nrf2

In 2002, Huang et al. found that Nrf2 could be phosphorylated at the Ser40 residue by protein kinase C (PKC), and the phosphorylation enhanced ARE-mediated transcription activity [58]. They further showed that Nrf2 was degraded by the ubiquitin-dependent pathway and that phosphorylation of Nrf2-Ser40 leads to an increase in its stability and subsequent transactivation activity [59]. In 2003, Numazawa et al. proposed that atypical PKC-iota (ι) is responsible for phosphorylation of Nrf2-Ser40 [60]. Cullinan et al. demonstrated that Nrf2 is a substrate of protein kinase RNA-activated (PKR)-like ER kinase (PERK). PERK-dependent phosphorylation of Nrf2 at the N-terminal region was shown to trigger dissociation of Nrf2/Keap1 complexes and subsequent Nrf2 nuclear import [61].

In 2006, Jain et al. showed that Fyn kinase can phosphorylate tyrosine (Tyr, Y) 576 (Mouse Tyr568) in the NES of Nrf2, and this phosphorylation is required for Crm1-mediated nuclear export and subsequent degradation of Nrf2 [62]. They further demonstrated that GSK3β acts as an upstream regulator of Fyn kinase in the control of the nuclear export of Nrf2 [63]. In this study, hydrogen peroxide (H_2_O_2_) activated GSK3β by phosphorylation of Tyr216 residue and the activated GSK3β phosphorylated Fyn kinase at threonine (Thr, T) residues, causing nuclear accumulation of Fyn. Fyn-dependent phosphorylation of Tyr576 (Mouse Tyr568) of Nrf2 led to nuclear export, ubiquitylation, and degradation of Nrf2.

Salazar et al. showed that GSK3β can directly phosphorylate Nrf2 and induce exclusion of Nrf2 from the nucleus [64]. Phosphoinositide 3-kinase (PI3K) and protein kinase B (PKB, Akt) increased the nuclear translocation of Nrf2 by inhibiting GSK3β kinase activity. In 2012, Rada et al. identified Ser344 and Ser347 (Mouse Ser335 and Ser338) of Nrf2 as the GSK3β-mediated phosphorylation sites [53]. These Ser residues are contained in one of the two binding motifs for β-TrCP.

Of the mitogen-activated protein kinases (MAPKs), p38 was first shown to phosphorylate Nrf2 and promote the association between Nrf2 and Keap1 proteins, thereby inhibiting the nuclear translocation of Nrf2 and transcriptional activity [65]. In contrast, extracellular signal-regulated kinase (ERK) and c-Jun N-terminal kinase (JNK) were shown to cause the release of Nrf2 from Keap1 in the cytosol, and translocation of Nrf2 into the nucleus [66]. Later, Ser215, Ser408, Ser558, Thr559, Ser577 of Nrf2 were proposed as the potential targets for MAPKs and their upstream kinases [67]. The mutation of those phosphorylation sites caused a moderate decrease in the transcriptional activity of Nrf2.

In 2007, Pi et al. proposed that Nrf2 is a substrate for phosphorylation by casein kinase 2 (CK2) although phosphorylation sites were not identified [68]. In the following year, Apopa et al. showed that CK2 phosphorylated Nrf2 at multiple sites in transcription activation domains, Neh4 and Neh5, which could be dephosphorylated by λ phosphatase [69]. Increased phosphorylation of these sites correlated with the nuclear translocation of Nrf2.

In 2012, Rada et al. showed that GSK3β can phosphorylate two Ser residues, Ser344 and Ser347 (Mouse Ser335 and Ser338) of Nrf2, which are one of two binding sites for β-TrCP [53].

In 2016, Joo et al. identified Ser558 (Ser550 in mouse) of Nrf2 as a target of adenosine monophosphate (AMP)-activated protein kinase (AMPK) [70]. AMPK activation caused nuclear accumulation of Nrf2 through phosphorylation of this Ser residue located in the canonical nuclear export signal. As AMPK can inhibit GSK3β, it was proposed that AMPK can directly phosphorylate Ser550 residue, and indirectly induce dephosphorylation of Tyr568 through inhibition of the GSK3β/Fyn pathway, resulting in the nuclear accumulation of Nrf2 for the ARE-driven gene transactivation.

As discussed above, the phosphorylation of Nrf2 at the Ser40 or a neighboring region by PKC, PERK, and CK2 can enhance nuclear translocation of Nrf2 and the transcription activity. AMPK-mediated phosphorylation of Ser558 also leads to Nrf2 activation. On the other hand, the phosphorylation of Tyr576, Ser344, Ser347, or near sites by Fyn kinase, GSK3β, and other enzymes can promote the nuclear export of Nrf2, and its sequestration by Keap1 or β-TrCP, ubiquitylation, and proteasomal degradation in the cytosol, which results in the reduced transcription activity. In addition, the PI3K/PKB (Akt) pathway and AMPK can indirectly promote the nuclear translocation and transcription activity of Nrf2 by inhibiting the GSK3β-mediated pathway. Of the MAPKs, p38 appears to moderately enhance nuclear export whereas ERK and JNK enhance nuclear import of Nrf2, although their target sites are not well-defined.

### 2.5. Acetylation of Nrf2

In 2009, Sun et al. first reported the acetylation of Nrf2 as a modulatory mechanism for ARE-dependent antioxidant response. They showed that a transcriptional coactivator, p300/CBP, acetylated multiple Lys residues (Lys 438, Lys 443, Lys 445; Lys 533, Lys 536, Lys 538) within the Neh1 DNA binding domain of Nrf2, which augmented binding of Nrf2 to ARE promoter of DNA.

A regulatory role of acetylation and deacetylation of Nrf2 for transcriptional activity was further shown by Kawai et al. who verified that CBP-mediated acetylation of Nrf2 increased the binding of Nrf2 to ARE in a gene promoter, and increased the transcription of target genes [71]. They also showed that sirtuin 1 (Sirt1, a class III HDAC) could induce deacetylation of Nrf2, using a molecular approach with the expression of heterologous Sirt1 and a dominant-negative Sirt1-H355A mutant, and a pharmacological approach with the Sirt1 inhibitors, EX-527 and nicotinamide, and a putative Sirt1 activator, resveratrol. The acetylation sites were identified to be Lys596 (Mouse Lys588) and Lys599 (Mouse Lys591) of Nrf2, and the acetylation increased nuclear localization of Nrf2, whereas deacetylation enhanced its cytoplasmic localization.

In 2017, Yang et al. showed that Sirt2, a cytoplasmic sirtuin (class III HDAC) physically interacts with Nrf2 and deacetylates the Lys 506 and Lys508 residues, leading to a reduced level of nuclear Nrf2 protein, reduced ferroportin 1 (FPN1) expression, and decreased cellular iron export [72]. They further showed that *Sirt2* deletion decreases cell viability under an iron deficiency condition. Moreover, livers from *Sirt2(-/-)* mice had increased protein levels of acetylated Nrf2 and FPN1, and decreased levels of iron, while these effects were reversed in *Sirt2(-/-)*/*Nrf2(-/-)* double knockout mice.

The acetylation of Nrf2 mainly occurred at Lys residues in the Neh1 and Neh3 domains where NES and nuclear localization signal (NLS) are located. Therefore, acetylation of Nrf2 at these regions is considered to play a regulatory role in the trafficking of Nrf2, enhancing its movement from the cytosol to the nucleus, in the opposite direction regulated by phosphorylation of Nrf2 at the Tyr576 (Mouse Tyr568) residue.

In a recent study by Ganner et al., p300 was shown to physically interact with Nrf2 and interfere with Nrf2-Keap1 complex formation [73]. The increase in acetyltransferase activity of p300 increased Nrf2 protein abundance and promoted Nrf2 nuclear localization. They proposed “a model whereby p300-mediated acetylation of Nrf2 causes dissociation of Nrf2 from Keap1, allowing Nrf2 to translocate to the nucleus and upregulate transcription of target genes, and eventually increasing survival under oxidative stress”.

### 2.6. Integrated Regulation of Nrf2 by Post-Translational Modifications and Protein–Protein Interactions

**Figure 4:**
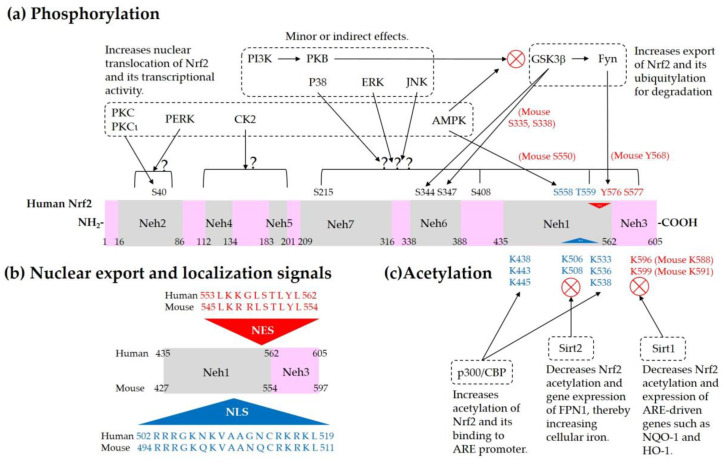
Regulation of Nrf2 through post-translational modifications. (**a**) Phosphorylation of Nrf2. Phosphorylation of Nrf2 at serine (Ser, S) 40 residue by protein kinase C (PKC) or atypical PKC-iota (ι), at Ser558 (Mouse Ser550) residue by AMP-activated protein kinase (AMPK), and at a N-terminal region by PKR-like ER kinase (PERK) can enhance nuclear translocation of Nrf2 and its transcription activity. On the other hand, phosphorylation of tyrosine (Tyr, Y) 576 (Mouse Tyr568) by a glycogen synthase kinase (GSK)3β/Fyn kinase pathway can promote the nuclear export of Nrf2 and its sequestration by Keap1, which results in the reduced transcription activity of Nrf2. The phosphoinositide 3-kinase (PI3K)/protein kinase B (PKB, Akt) pathway and AMPK can indirectly promote the transcription activity of Nrf2 by inhibition of GSK3β activity. GSK3β can directly phosphorylate Ser344 and Ser347 (Mouse Ser335 and Ser338) in the ^343^DSGIS^347^ motif of Nrf2, enhance the binding to β-TrCP, and thus increase ubiquitylation and proteasomal degradation of Nrf2. Mitogen-activated protein kinases (MAPKs), such as p38, extracellular signal-regulated kinase (ERK), and c-Jun N-terminal kinase (JNK), can phosphorylate Ser or threonine (Thr, T) residues of Nrf2 directly or indirectly, and modulate Nrf2 activity either negatively or positively, and only moderately in both cases. (**b**) Nuclear export signal (NES) and nuclear localization signal (NLS). Human and mouse sequences are aligned for comparison. (**c**) Acetylation of Nrf2. Acetylation of lysine (Lys, K) residues in the NES or NLS of Nrf2 resulting from increased levels of p300/CBP or decreased level of Sirt1 and Sirt2 can prevent nuclear export and ubiquitylation-mediated degradation of Nrf2, and increase nuclear accumulation and transcriptional activity of Nrf2.

**Figure 5:**
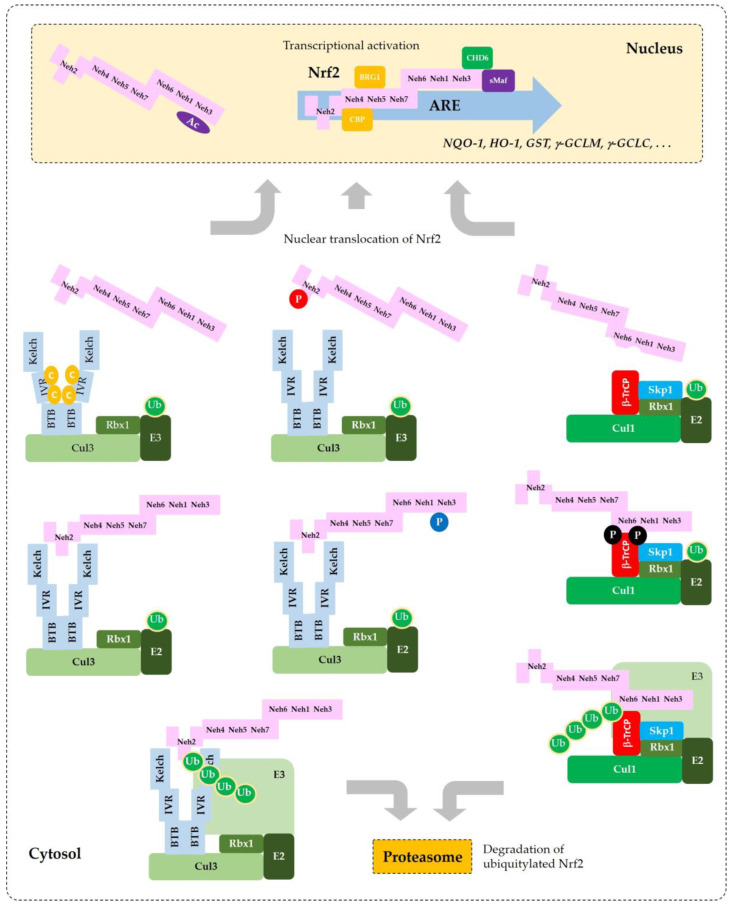
Coordinated regulation of Nrf2 by its post-translation modification in conjunction with its sequestration by Keap1 or β-TrCP. This schematic represents a “simplified” model for multiple states of Nrf2 that interact with Keap1 or β-TrCP with different binding affinities. Phosphorylation of Nrf2 at Tyr576 (P in blue circle) by Fyn kinase can enhance the interaction between Nrf2 and Keap1, which results in increased ubiquitylation (Ub in green circles) and subsequent proteasomal degradation of Nrf2. Phosphorylation of Nrf2 at Ser40 (P in red circle) by PKC, or the oxidative and/or chemical modification of Keap1 at Cys residues (C in yellow circles) in the IVR can weaken the interaction between Nrf2 and Keap1, which leads to an increase of Nrf2 that is released from Keap1 and enters the nucleus to transcriptionally activate ARE-driven gene expression. Phosphorylation of Nrf2 at Ser344 and Ser347 (P in black circles) by GSK3β enhances the interaction between Nrf2 and β-TrCP, which results in increased ubiquitylation and subsequent proteasomal degradation of Nrf2. If GSK3β is inhibited by PKB (Akt) or other enzymes, these Ser residues are dephosphorylated and Nrf2 can be released from β-TrCP, which allows nuclear translocation of Nrf2 and transcriptional activation. Acetylation of Lys residues (Ac in purple oval) in the NES of Nrf2 by CBP/p300 can enhance nuclear import and accumulation of Nrf2.

Current understanding of the regulation of Nrf2 by phosphorylation and acetylation is illustrated in Figure 4. In Figure 5, a simplified model for coordinated regulation of Nrf2 by its post-translational modification and its interaction with Keap1 or β-TrCP is schematically illustrated. It is apparently complicated, and it will become even more complicated if other known and currently unknown contributors to the flux of Nrf2 are all included [74].

## 3. Plant-Derived Natural Nrf2 Modulators for Skin Protection

### 3.1. Sulforaphane

**Figure 6:**
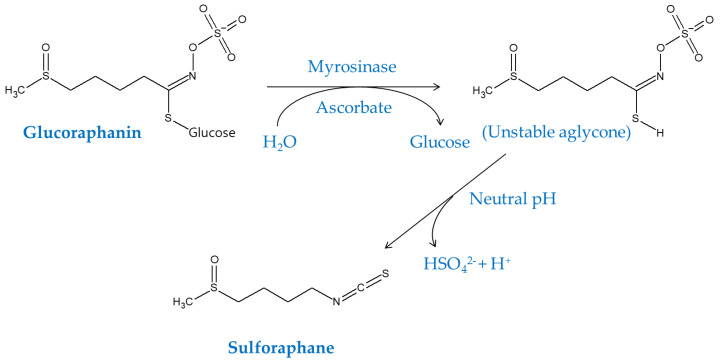
Bioconversion of glucoraphanin to sulforaphane, an isothiocyanate compound, by enzymatic hydrolysis by myrosinase, which requires ascorbate as a cofactor. The reaction can also produce trace products with nitrile or thiocyanate moieties.

Sulforaphane is a naturally occurring organosulfur compound produced by cruciferous vegetables such as broccoli and has a property to activate Nrf2-dependent pathways [75]. Sulforaphane can modify critical Cys residues of Keap1, which lead to dissociation of Nrf2 from the Keap1/Nrf2 complex and its nuclear translocation to induce ARE-driven gene expression [76,77]. Sulforaphane can be produced from its precursor glucoraphanin by the action of myrosinase (EC 3.2.1.147, thioglucoside glucohydrolase) that requires ascorbate as a cofactor [78], as shown in Figure 6. Pure sulforaphane compound or foods rich in sulforaphane and/or glucoraphanin have been tested in various disease models [79].

Xu et al. tested sulforaphane against skin carcinogenesis induced by the combined treatment of a chemical carcinogen, 7,12-dimethylbenz(a)anthracene (DMBA), and a tumor promoter, 12-*O*-Tetradecanoylphorbol-13-acetate (TPA) in *Nrf2(-/-)* knockout and wild type C57BL/6 mice [80]. In this classical 2-stage carcinogenesis model, the incidence of skin tumors, and tumor numbers per mouse were higher in *Nrf2(-/-)* knockout mice as compared with wild type mice. Topical application of sulforaphane prior to the chemical treatments decreased the incidence of skin tumor compared with the control group in the wild type mice, and such chemoprotective effect of sulforaphane was not seen in the *Nrf2(-/-)* knockout mice, implicating that that *Nrf2(-/-)* knockout mice are more susceptible to chemically induced skin tumorigenesis and that Nrf2 is involved in the chemoprevention by sulforaphane.

Dinkova-Kostova et al. tested a standardized myrosinase-hydrolyzed broccoli sprout extract containing sulforaphane in SKH-1 hairless mice [81]. The topical application of the extract increased glutathione and NQO-1 levels in the skin tissues. The extract inhibited the skin carcinogenesis induced by UV-radiation, as determined by tumor incidence, multiplicity, and overall tumor burden in mice, supporting the protective effects of sulforaphane against skin tumor formation after exposure to UV radiation.

Epidermolysis bullosa simplex (EBS) is an inherited disease that accompanies the loss of epidermal integrity after mechanical trauma. Kerns et al. showed that sulforaphane elicited distinct transcriptional programs, inducing expression of keratins 16 and 17 and thereby alleviating the blistering in keratin 14 null neonatal mice, an EBS model [82]. The induction of keratin 16 by sulforaphane was partly attenuated in *Nrf2(-/-)* SKH-1 hairless mice, whereas keratin 17 induction was not affected [83]. Therefore, it was suggested that both Nrf2-dependent and -independent pathways are involved in alterations in keratin 16 and 17 levels in sulforaphane-treated epidermis.

In a study by Saw et al. a single dose of UVB caused skin inflammation in both wild type and *Nrf2(-/-)* knockout C57BL/6 mice [84]. The inflammation degrees returned to the basal level in wild type mice to a greater extent than that in knockout mice. In addition, sulforaphane treatment reduced the number of sunburn cells back to the basal level within 8 d after UVB irradiation in wild type, but not in *Nrf2(-/-)* knockout mice. Inflammatory biomarkers (IL-1β and IL-6) and apoptotic cells were significantly higher in knockout mice than in wild type mice [84].

In healthy human subjects, topical applications of the extracts delivering sulforaphane attenuated the degree of solar-simulated UV radiation-induced skin erythema, a surrogate endpoint for cutaneous damage and skin cancer risk [85]. Genetic or pharmacologic Nrf2 activation lowered the expression of the pro-inflammatory factors, such as interleukin (IL)-6 and IL-1β, and cyclooxygenase-2 (COX-2) after acute exposure of mice to UV radiation [85].

Chawalitpong et al. reported that long-term intake of a glucoraphanin-enriched kale diet lowered the senescence grading score and suppressed the thinning of the dorsal skin layer in senescence-accelerated mouse prone 1 (SAMP1) mice by enhancing antioxidant capacity via the Nrf2 pathway. The diet increased collagen production via the transforming growth factor (TGF)-β receptor (TβR) 2/similarity to the Drosophila gene mothers against decapentaplegic (MAD) 3 (Smad3) pathway [86].

Many previous studies have shown that sulforaphane or its precursors can have anticancer, anti-inflammatory, and antiaging effects in the skin. However, regarding the use of sulforaphane as an anticancer agent, great caution is needed because it could block the T cell-mediated immune response, which is important for immune surveillance of tumors [87].

### 3.2. Terpenoids

**Figure 7:**
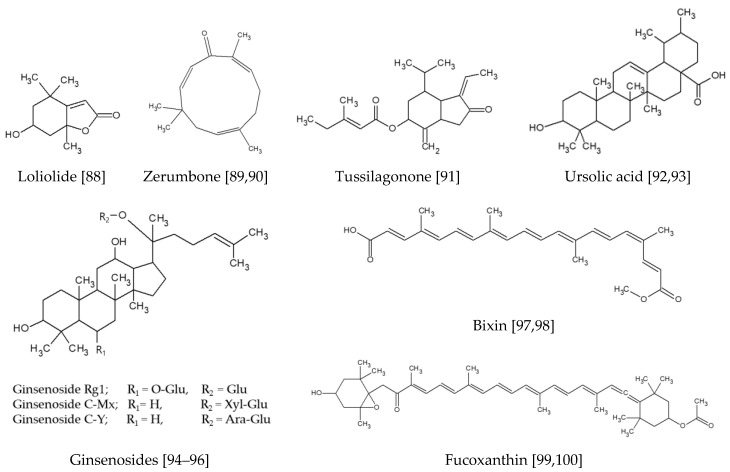
Terpenoid compounds activating the Nrf2-mediated pathway [88,89,90,91,92,93,94,95,96,97,98,99,100].

The terpenoids are a class of phytochemicals derived from the 5-carbon compound isoprene, and can be classified according to the number of isoprene units; monoterpenoids, two isoprene units; sesquiterpenoids, three units; diterpenoids, four units; triterpenoids, six units; tetraterpenoids, eight units. Figure 7 shows chemical structures of terpenoid compounds discussed in this section.

Loliolide, a monoterpenoid hydroxylactone contained in *Prasiola japonica*, suppressed gene expression of matrix metalloproteinases (*MMP*s), *Nrf2*, and *HO-1* in HaCaT Keratinocytes stimulated by H_2_O_2_ [88].

Zerumbone, a sesquiterpenoid that can be found in *Zingiber zerumbet*, suppressed UVA-induced ROS production, DNA damages, and apoptotic death of HaCaT keratinocytes through a Nrf2-dependent mechanism [89]. It activated Nrf2/ARE signaling by the p38 MAPK, PI3K/PKB (Akt), and PKC signaling cascades, and induced expression of *HO-1* and *γ-GCLC* genes. Its topical treatment to nude mice ameliorated UVA cytotoxicity via increased nuclear localization of Nrf2 and expression of Nrf2-dependent antioxidant genes in the skin. In human skin fibroblasts, zerumbone inhibited UVA-induced ROS generation through a Nrf2/ARE-mediated defense mechanism [90]. It reduced MMP-1 expression and senescence associated-β-galactosidase (SA-β-Gal) activities stimulated by UVA. Tussilagonone, a sesquiterpenoid contained in *Tussilago farfara*, suppressed expression of psoriasis-related inflammatory genes and keratinocyte hyperproliferation through activation of Nrf2 [91]. Its topical application ameliorated phenotypical changes in the mouse model of imiquimod-induced psoriasis-like dermatitis.

Ursolic acid, a triterpenoid found in blueberries, cranberries, and apple peels, inhibited cellular transformation by TPA through the Nrf2-mediated expression of detoxifying/antioxidant enzymes HO-1, NQO-1, and UGT1A1 in mouse epidermal JB6 P+ cells [92]. It reduced the expression of epigenetic modifying enzymes, such as DNA methyltransferases (DNMTs) and histone deacetylases (HDACs), thereby causing demethylation of the first 15 CpG sites of the Nrf2 promoter region, which leads to enhanced expression of Nrf2. Ursolic acid stimulated Nrf2 and its upstream/downstream genes, anti-inflammatory and cell cycle regulatory genes, and exhibited chemopreventive effects against nonmelanoma skin cancer induced by UVB radiation in SKH-1 hairless mice [93].

Several ginsenosides derived from *Panax ginseng* were shown to enhance the defense capacity of HaCaT keratinocytes against UV radiation. Ginsenoside Rg1 restored glucocorticoid receptor (GR) that had been depleted by UVB irradiation, and reduced reactive oxygen species (ROS) generation and increased HDAC2 expression by a Nrf2-dependent mechanism. It potentiated the anti-inflammatory effects of dexamethasone in UVB-irradiated Balb/c mouse skin through the Nrf2/HDAC2 pathway [94]. Ginsenoside C-Mx alleviated UVB-induced ROS, MMP-1, and IL-6 expression in normal human dermal fibroblasts, while accelerating TGF-β and procollagen type I secretion. It increased the expression of HO-1 and NQO-1 by enhancing the nuclear accumulation of Nrf2 [95]. Ginsenoside C-Y also blocked UVB-exposed ROS, restricted MMP-1 production, and promoted procollagen type I synthesis. Ginsenoside C-Y increased TGF-β1 level and fortified Nrf2 nuclear translocation and restricted AP-1 and MAPKs phosphorylation [96].

Bixin, an apocarotenoid derived from *Bixa orellana*, activated Nrf2 through the critical Cys151 sensor residue in Keap1, orchestrating a cytoprotective program in human keratinocytes as demonstrated by antioxidant gene expression array analysis [97]. Systemic administration of bixin suppressed epidermal oxidative DNA damage and inflammatory responses in wild type mice but not in *Nrf2(-/-)* knockout SKH-1 mice [97]. Topical bixin treatment suppressed acute epidermal hyperproliferation and oxidative DNA damage induced by UV in wild type SKH-1 mice but not in *Nrf2(-/-)* knockout mice, and retarded hair graying induced by combined treatment of psoralen plus UVA in wild type C57BL/6J mice but not in *Nrf2(-/-)* knockout mice [98]. Thus, bixin can protect against photo-oxidative damages of skin or hair graying through Nrf2 activation. Fucoxanthin, a carotenoid found in various microalgae and seaweeds, suppressed TPA-induced transformation of mouse skin JB6 P+ cells [99]. It increased the messenger ribonucleic acid (mRNA) and protein levels of Nrf2, ARE-luciferase activity, and expression of Nrf2 downstream genes. It decreased the methylation of the Nrf2 promoter region and reduced DNMT activity without any effects on HDAC activity. Fucoxanthin-containing cream ameliorated TPA-induced hyperplasia, by reducing mouse skin edema, epidermal thickness, myeloperoxidase (MPO) activity, and COX-2 expression [100]. The cream downregulated COX-2 and inducible nitric oxide synthase (iNOS) and upregulated HO-1 via the Nrf2 pathway.

### 3.3. Flavonoids and Stilbenoids

**Figure 8:**
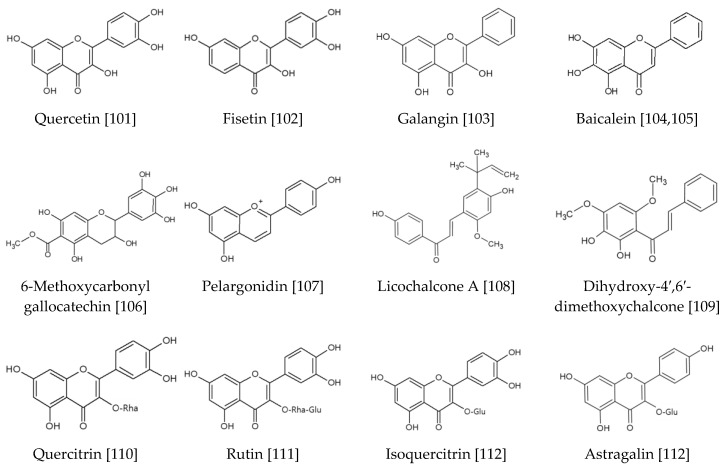

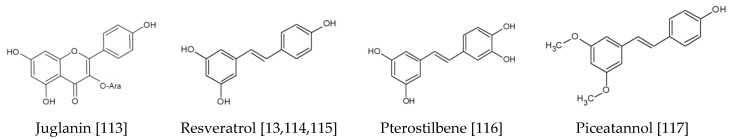
Flavonoid and stilbenoid compounds activating the Nrf2-mediated pathway [13,101,102,103,104,105,106,107,108,109,110,111,112,113,114,115,116,117].

In this section flavonoids and stilbenoids with Nrf2 modulating activity are discussed. Chemical structures of these compounds are shown in Figure 8.

Of the flavonoid aglycones, quercetin attenuated atopic dermatitis-like lesion induced by the house dust mite extract in NC/Nga transgenic mice [101]. The compound downregulated high-mobility group box-1 (HMGB-1) cascade signaling and upregulated nuclear Nrf2. Fisetin reduced ROS production and death of HaCaT keratinocytes by H_2_O_2_ [102]. It inhibited the production of Nitric oxide (NO), Prostaglandin (PG)-E2, IL-1β, IL-6, expression of iNOS and COX-2, and activation of nuclear factor kappa-light-chain-enhancer of activated B cells (NF-κB) induced by tumor necrosis factor-α (TNF-α). It induced Nrf2 translocation to the nuclei and increased HO-1 expression. HO-1 silencing RNAs reversed the cytoprotective, antioxidant, and anti-inflammatory effects of fisetin. Galangin was shown to protect human keratinocytes by activating ERK/Akt/Nrf2, leading to elevated expression of glutathione-synthesizing enzymes, such as γ-GCLC and glutathione synthetase [103]. Baicalein from *Scutellaria baicalensis* inhibited H_2_O_2_-induced cytotoxicity and apoptosis in human vitiligo melanocytes (PIG3V) by promoting Nrf2 nuclear translocation, and the expression of Nrf2 and HO-1 [104]. Baicalein also attenuated the toxic effects of benzo[a]pyrene in human epidermal keratinocytes and HaCaT cells via inhibition of the AhR-cytochrome P450 1A1 pathway, and activation of a Nrf2/HO-1 pathway [105]. 6-Methoxycarbonyl gallocatechin from Anhua dark tea suppressed the activation of signal transduction and activation of transcription 1 (STAT1), and expression of inflammatory cytokines, and activated the Nrf2 pathway to protect cells from ROS production in UVB exposed HaCaT keratinocytes [106]. Pelargonidin, an anthocyanidin compound, blocked TPA-induced transformation of mouse epidermal JB6 P+ cells, and this effect was attributed to its activation of the Nrf2-ARE signaling pathway through epigenetic mechanisms [107]. Licochalcone A protected primary human fibroblasts from UVA-induced oxidative stress through the expression of cytoprotective phase II enzymes, such as HO-1 and γ-GCLM [108]. Topically applied licochalcone A-containing lotion lowered ROS levels in human skin. 2′, 3′-Dihydroxy-4′, 6′-dimethoxychalcone, from green perilla, suppressed UV-A radiation-induced ROS production and rescued the viability of HaCaT keratinocytes by enhancing HO-1 expression [109].

Of the flavonoid glycosides, quercitrin blocked TPA-induced neoplastic transformation in mouse JB6 P+ cells [110]. It downregulated transactivation of AP-1 and NF-κB, and activated the Nrf2 pathway. Rutin attenuated inflammatory response, ROS generation, lipid peroxidation, and protein modifications induced by UV in human fibroblasts [111]. It enhanced Nrf2 expression and the activity/levels of antioxidants. It also prevented the changes in expression levels of the endocannabinoid system and apoptotic mediators. Isoquercitrin and astragalin activated Nrf2 and heat-shock response transcription elements (HSE) that resulted in the induction of HO-1 and heat shock protein 70 (HSP70), respectively, in human dermal fibroblasts and epidermal keratinocytes [112]. These compounds inhibited apoptosis due to UVB irradiation. Juglanin attenuated UVB-triggered oxidative stress and inflammatory responses in hairless mice through enhancement of Nrf2 activity and suppression of MAPKs and NF-κB signaling pathways [113].

Of the stilbenoid compounds, resveratrol attenuated UVA-induced oxidative stress and death of HaCaT keratinocytes [114]. It decreased Keap1 protein level and increased Nrf2 protein level, facilitating Nrf2 accumulation in the nucleus, which led to enhanced expression of antioxidant enzymes. Resveratrol induced mild Nrf2-specific gene expression reprogramming, resulting in a quantitative reduction of the cellular redox environment in primary human epidermal keratinocytes [13]. Ethanol-induced ROS generation was attenuated in resveratrol-pretreated cells, suggesting that minor oxidative triggering by resveratrol can shift cellular defense towards a more reductive state to improve physiological resistance to severe oxidative stress. Oral administration of either grape peel extract or resveratrol in mice attenuated epidermal thickening and wrinkle formation in skin exposed to UVB through activation of the Nrf2/HO-1 signaling pathway [115]. Pterostilbene, but not resveratrol, prevented chronic UVB-induced skin carcinogenesis through enhancing antioxidant defenses, such as catalase, SOD, and GPX activities, and mitigating oxidative damages to DNA, protein, and lipids in the skin of SKH-1 hairless mice [116]. It activated Nrf2-dependent antioxidant response in cultured HaCaT keratinocytes. Piceatannol inhibited *Propionibacterium acnes*-induced HaCaT cell proliferation and migration by activating the antioxidant Nrf2 pathway and inhibiting the inflammatory NF-κB pathway [117].

### 3.4. Other Compounds Derived from Land Plants

**Figure 9:**
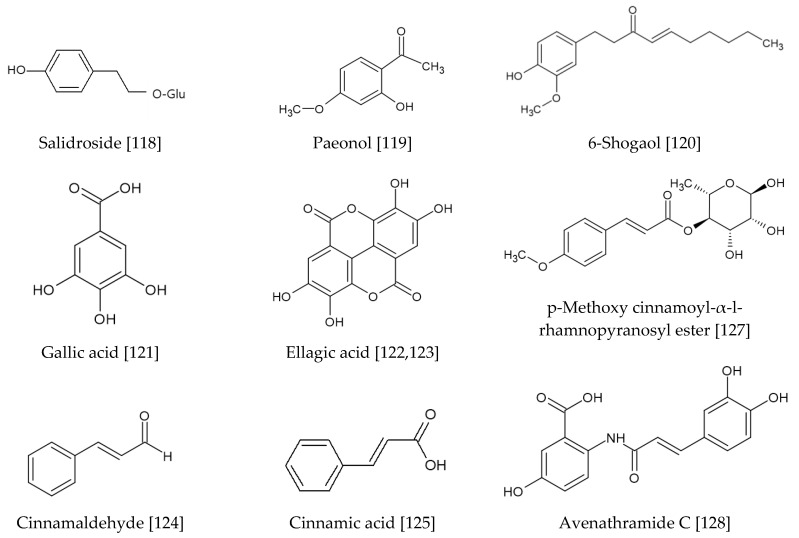

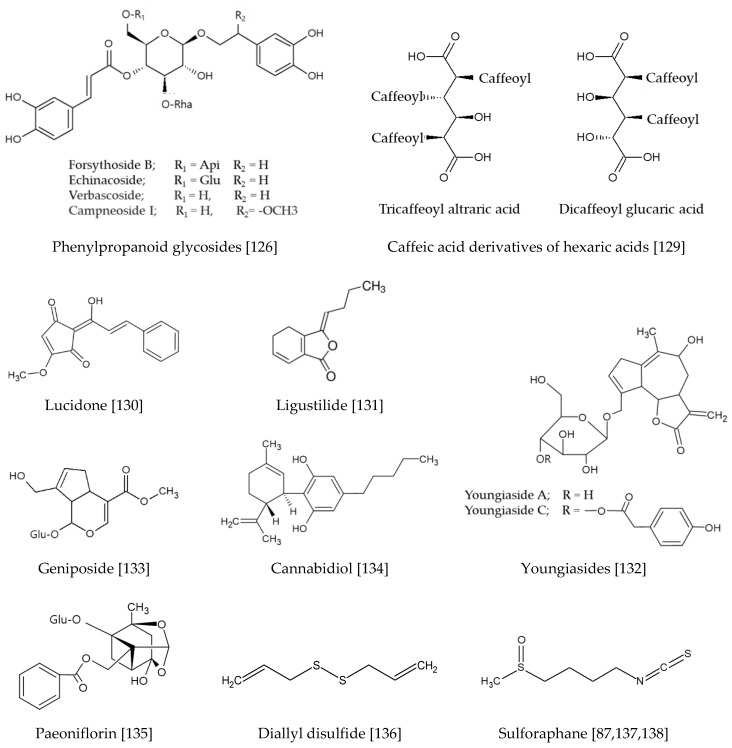
Various natural products modulating the Nrf2-mediated pathway [87,118,119,120,121,122,123,124,125,126,127,128,129,130,131,132,133,134,135,136,137,138]. Gallic acid inhibits the Nrf2-mediated pathway and all other compounds activate this pathway.

Various compounds shown in Figure 9 are discussed in this section. Of simple phenol compounds, salidroside, a glucoside of tyrosol found in *Rhodiola rosea*, increased nuclear accumulation of Nrf2, and expression of NQO-1 and HO-1 in HaCaT keratinocytes [118]. It reduced ROS production and oxidative damages in UV-irradiated cells. Its oral administration decreased apoptotic sunburn cells and 8-hydroxy deoxyguanosine-positive epidermal cells in the skin of guinea pigs irradiated with UVB. Paeonol derived from *Paeonia suffruticosa* inhibited UVB-induced phosphorylation of MAPKs and AP-1 involved in the degradation of procollagen type I in keratinocytes [119]. On the other hand, it increased NQO-1 and HO-1 expression, by enhancing the nuclear accumulation of Nrf2 in hairless mice. In vivo, the topical application of an extract of *Paeonia suffruticosa* and paeonol attenuated UVB-induced MMP-1 expression and promoted procollagen type I production in hairless mice. 6-Shogaol, a bioactive compound from ginger, attenuated H_2_O_2_- or rhododendrol-induced cell death and oxidative stress of human epidermal melanocytes through the induction of Nrf2-mediated expression of HO-1 and NQO-1 [120].

Gallic acid, a simple phenolic acid found in many plants such as *Paeoniae Rubra*, reduced psoriasis area, severity index scores, and epidermal hyperplasia of psoriasis-like disease mice [121]. It decreased expression of keratin 16 and 17 by downregulating Nrf2 activity. Ellagic acid, a polyphenolic constituent of plants, attenuated ROS generation, oxidative damages, apoptotic death of HaCaT keratinocytes exposed to UVA [122]. It downregulated Keap1 and activated Nrf2, thereby increasing the expression of HO-1 and SOD. Ellagic acid also suppressed UVB-induced ROS production and MMP-2 expression in human dermal fibroblasts [123]. It restored total glutathione contents, SOD activity levels, and Nrf2 levels diminished by UV-B irradiation.

Cinnamaldehyde, a major component of *Cinnamomum cassia*, inhibited the AhR pathway, activated the Nrf2/HO-1 pathway, and alleviated ROS production induced by benzo[a]pyrene in normal human epidermal keratinocytes and HaCaT cells [124]. Its inhibition of AhR signaling and the activation of antioxidant activity operated in a mutually independent manner. Cinnamic acid, a representative phenylpropanoid compound, ameliorated UVA-induced cytotoxicity and inhibited ROS production, expression of MMP-1/-3, and degradation of type I procollagen in human foreskin fibroblasts (Hs68) [125]. It increased nuclear translocation of Nrf2 through PKC, AMPK, CKII or ROS signaling cascades, and induced HO-1 and γ-GCLC expressions. Its bioactivities are abolished by Nrf2 silencing RNAs. It suppressed MMP-1/-3 activation and maintained type I procollagen levels of the skin tissue in UVA-irradiated nude mice.

The phenylpropanoid glycosides, forsythoside and echinacoside, induced nuclear translocation of Nrf2 protein and reduced nuclear protein levels of Bach1, thereby increasing expression of Phase II enzymes, such as HO-1, in HaCaT keratinocytes (verbascoside and campneoside were less active) [126]. Their aglycone, hydroxytyrosol, showed similar activity. p-Methoxy cinnamoyl-α-l-rhamnopyranosyl ester, a phenylpropanoid compound isolated from *Scrophularia buergeriana*, induced Nrf2 activity and increased expression of HO-1 in HaCaT keratinocytes. It enhanced Nrf2 stability by blocking proteasomal degradation [127]. Avenathramide C, found exclusively in oats, protected cells against H_2_O_2_ or TNF-α-induced oxidative stress and inflammatory response through NF-κB inhibition and Nrf2/HO-1 activation in normal human skin fibroblasts [128]. The derivatives of hexaric acids conjugated with several caffeic acid moieties isolated from *Galinsoga parviflora*, tricaffeoyl altraric acid and dicaffeoyl glucaric acid, inhibited ROS formation, increased glutathione level, and rescued viability of normal human dermal fibroblasts exposed to UVA [129]. These compounds activated the Nrf2 and increased HO-1 expression.

Lucidone, a naturally occurring cyclopentenedione in plants such as *Lindera erythrocarpa*, increased cell viability and suppressed ROS generation, lipid peroxidation, and DNA damage induced by 2,2′-azobis (2-amidinopropane) dihydrochloride (AAPH), by increasing Nrf2-mediated expression of HO-1 in HaCaT keratinocytes [130]. It also inhibited AAPH-induced COX-2 expression and PGE_2_ production. Ligustilide, a phthalide derivative found in *Cnidium officinale* and *Angelica acutiloba*, inhibited UVB-induced ROS production in normal human epidermal keratinocytes, by a Nrf2 and HO-1-dependent mechanism [131]. It also reduced UVB-induced production inflammatory mediators, such as IL-6, IL-8, and monocyte chemoattractant protein-1 (MCP-1), by suppressing the NF-κB pathway. Youngiaside A and C, isolated from *Youngia denticulatum*, decreased MMP-1 expression in HaCaT keratinocytes and human dermal fibroblasts, by AMPK and Nrf2-dependent mechanisms [132]. These compounds increased antioxidant enzyme expression thereby downregulating UVB-induced ROS production, MAPKs expression, and AP-1 signaling. They also inhibited NF-κB signaling and suppressed expression of pro-inflammatory mediators.

Geniposide (genipin 1-glucoside), an iridoid glycoside from *Gardenia jasminoide*, attenuated production of ROS and expression of proMMP-2 induced by UVB-irradiation in human dermal fibroblasts [133]. It upregulated Nrf2 level and total SOD activity, and total glutathione content under UV-B irradiation. Cannabidiol, a phytocannabinoid from *Cannabis sativa*, enhanced the activity of antioxidant enzymes and reduced lipid peroxidation in UV-irradiated keratinocytes [134]. It showed antioxidant activity through Nrf2 activation, and anti-inflammatory activity through NF-κB inhibition. Paeoniflorin, a phytochemical in *Paeonia lactiflora*, attenuated UVA-induced photodamages via activation of Nrf2/HO-1/NQO-1 signaling pathway in human dermal fibroblasts [135].

Diallyl disulfide, a major garlic derivative, attenuated skin tumor incidence and multiplicity in a carcinogenesis model induced by cutaneous application of DMBA and subsequent TPA [136]. It upregulated a bunch of antioxidant enzymes activities and the nuclear accumulation of Nrf2. Its preventive effects against skin carcinogenesis were reversed in *Nrf2(-/-)* knockout mice. Sulforaphane is discussed in Section 3.1.

### 3.5. Marine Natural Products

**Figure 10:**
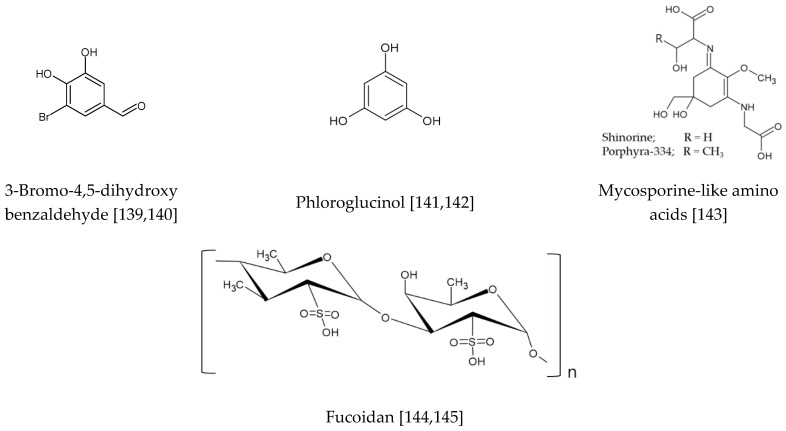
Marine natural products activating the Nrf2-mediated pathway [139,140,141,142,143,144,145].

Marine natural products shown in Figure 10 are discussed in this section. 3-Bromo-4,5-dihydroxy benzaldehyde, a natural bromophenol found in marine red algae and seaweeds, increased the production of glutathione and the expression levels of glutathione synthesizing enzymes in HaCaT keratinocytes [139]. It activated Nrf2 via an ERK and PKB-dependent mechanism and established cellular protection against oxidative stress via a Nrf2-mediated pathway. It also activated Nrf2 signaling cascades, upregulated HO-1, and protected cells from H_2_O_2_- and UVB-induced oxidative damage in HaCaT keratinocytes [140]. Phloroglucinol, a major component of phlorotannins abundantly present in marine brown alga species, protected HaCaT keratinocytes against H_2_O_2_-induced DNA damage and attenuated apoptosis through activating the Nrf2/HO-1 signaling pathway [141]. It also restored OGG-1 expression via a Nrf2-mediated pathway, which resulted in decreased levels of 8-oxoguanine in UVB-exposed HaCaT keratinocytes [142].

Porphyra-334 and shinorine are mycosporine-like amino acids found in cyanobacteria and marine algae. These compounds activated the Nrf2 pathway by binding to Keap1, causing dissociation of Nrf2 from keap1, and thereby increased expression of ARE-driven genes in primary skin fibroblasts [143].

Fucoidan from *Sargassum horneri* reduced ROS production and rescued the viability of UVB-exposed HaCaT cells by enhancing a Nrf2/HO-1 signaling pathway [144]. Fucoidan from *Sargassum siliquastrum* also increased Nrf2-mediated HO-1 expression and suppressed ROS production and mitochondria-mediated apoptosis in UVB-exposed HaCaT keratinocytes [145].

## 4. Discussion

Through this review, we discussed “historically” notable studies on the discovery and mechanism of action of the Nrf2-mediated defense pathway, as well as recent studies on using natural substances that activate this pathway to enhance the defense capacity of skin cells. In the cytosol at basal state, Keap1 and β-TrCP sequester Nrf2 and promote its degradation, thereby keeping the progression of the Nrf2-mediated defense pathway low. These processes are finely tuned through various post-translational modifications of Nrf2 and interactions with other protein factors. When ROS or electrophilic substances cause modifications in the reactive Cys residues of Keap1, or when dephosphorylation of the regulatory Ser residue in the Neh6 domain of Nrf2 is induced by cell survival signals such as the PI3K/Akt pathway that inhibits GSK3β activity, Nrf2 is released from Keap1 or β-TrCP and can enter the nucleus.

There were inconsistent reports regarding the role of p38 MAPK in the regulation of Nrf2. Keum et al. showed that sulforaphane activated the Nrf2/ARE pathway by involving the downregulation of p38 MAPK activity in HepG2 cells [65]. Yang et al. showed that zerumbone activated Nrf2/ARE signaling by involving the upregulation of p38 MAPK activity in HaCaT cells [89]. The reason for this discrepancy is currently unknown. It might be associated with the involvement of different isoforms of p38 MAPK, such as p38α, p38β, p38γ, and p38δ that are differentially expressed depending on cell types and physiological contexts [146]. Another possibility is that p38 MAPK may act as an indirect modulator of Nrf2 that shows variable effects depending on other major signaling pathways. Nonetheless, p38 and other MAPKs may not be main players in the regulation of Nrf2 activity because their activity displays only moderate effects on the ARE-driven gene expression. Rather, PKC, Fyn kinase, and GSK3β can play a critical role in the regulation of the Nrf2-mediated pathway.

In the nucleus, Nrf2 forms a heterodimer with sMaf, binds to the ARE promoter of DNA, and shows transcriptional activity in cooperation with several coactivators, such as CBP, BRG1 and CHD6. Nrf2 implements a gene expression program that meets the physiological needs of cells through cooperative action with other transcription factors in order to differentially regulate the expression of various ARE-driven genes [11,12]. The gene expression of *Nrf2* can be self-regulated through a positive feedback loop [147]. It is also subject to regulation by epigenetic mechanisms involving DNA methylation, histone modification, and microRNAs (miRs) [148]. miRs are recognized to play a critical role in the regulation of the Nrf2 pathway. miRs, such as miR-144, miR-28, and miR-93, diminish the Nrf2 signaling pathway via various mechanisms [149,150,151], whereas miR-200a and miR-7 activate Nrf2 signaling pathway by targeting keap1 [151,152]. There is a comprehensive review on miR-mediated regulation of Nrf2 activity [153].

There is still no complete answer to the question of whether multiple diseases can be prevented and treated by activating the Nrf2-mediated defense pathway, which was mentioned in the introduction. This is because living organisms can survive even if Nrf2 is deficient [10,25], and if Nrf2 is excessively activated due to Keap1 deficiency, it is rather fatal [154]. Activating Nrf2 can prevent cancer, but there is a dark side that Nrf2 activation can accelerate the progression of cancer once it has already developed [155]. In addition, activation of Nrf2 can reduce immune surveillance against cancer cells by inhibiting the function of immune cells, so Nrf2 should be considered as a double-edged sword that is more dangerous if used incorrectly [87].

The epidermis of the psoriatic lesion is characterized by thickening, scaling, and erythema, and it accompanies Nrf2-dependent overexpression of keratin 6, 16, and 17 [156]. Consistently, Nrf2 protein levels were higher in the epidermis of skin biopsy samples from psoriatic patients than those from healthy control subjects [156]. In addition, Nrf2 mRNA and protein levels were markedly increased in psoriatic lesion epidermis compared with the normal control samples from the same patient [156]. This is the virtually opposite situation observed in EBS [82]. When the keratin is excessively produced, psoriasis occurs [156], and if it is not produced well, EBS occurs [82].

Sulforaphane was shown to promote the synthesis of keratin through Nrf2 activation, and as a result, EBS symptoms were relieved [82]. On the other hand, gallic acid was shown to decrease the synthesis of keratin through suppression of Nrf2, and as a result, the symptoms of psoriasis were alleviated [121]. These studies suggest that, if the activity of Nrf2 is insufficient or excessive in skin diseases, it is possible to alleviate the disease by promoting or inhibiting the Nrf2-mediated pathway. Thus, both activators and inhibitors of Nrf2 may be useful in the prevention and treatment of skin diseases, and it will be important to apply them appropriately to the needs of the disease.

Tussilagonone was shown to alleviate imiquimod-induced psoriasis-like dermatitis through the activation of Nrf2 [91]. In addition, sulforaphane, a Nrf2 activator, enhanced recovery from the psoriasiform process in severe combined immunodeficiency (SCID) mice transplanted with normal human skin, and subsequently injected with IL-2-stimulated human psoriatic natural killer-like cells [157]. Thus, these Nrf2 activators are considered to alleviate psoriasis-like dermatitis through suppression of inflammatory reactions rather than to exacerbate the symptom by promoting excessive keratin synthesis.

Quercetin attenuated atopic dermatitis-like lesion induced by the house dust mite extract in NC/Nga transgenic mice by involving activation of the Nrf2-mediated pathway [101]. Sulforaphane showed anti-inflammatory effects in an atopic dermatitis mouse model induced by 2,4-dinitrochlorobenzene (DNCB) through the activation of the Nrf2/HO-1 axis [158]. Baicalein attenuated apoptosis of human vitiligo melanocytes (PIG3V) by activating the Nrf2/HO-1 pathway [104]. Galangin that activates the ERK/Akt/Nrf2 pathway [103], rescued melanocytes in a vitiligo model of C57BL/6 mice induced by hydroquinone [159]. Thus, natural Nrf2 modulators have the therapeutic potential to alleviate atopic dermatitis and vitiligo vulgaris.

Ideally, it is necessary to change the activity of Nrf2 dynamically and appropriately in the cell according to the changes in the microenvironment around the cell. This is a technically very difficult task but may not be impossible if we exploit multimodal approaches taking accounts of multilayered regulatory mechanisms for Nrf2 activity. Further efforts are also required to develop drugs that selectively modulate Nrf2. Sulforaphane, which is well known as an activator of Nrf2, also has a variety of other biological activities, so it can affect the physiology and pathology of a living body through a Nrf2-dependent and -independent mechanism [137,138]. Optimal candidate drugs should be identified through comparative studies on the structure–activity relationship of many known and currently unknown substances with Nrf2 modulating activity.

Terrestrial and marine plants provide a source of various bioactive substances [160]. As discussed in this review, many studies have shown that terpenoids (including mono-, sesqui-, and triterpenoids, saponins, and carotenoids), phenolic compounds (flavonoids, stilbenoids, phenols, phenolic acids, and phenylpropanoids), and other compounds derived from land plants and various natural products derived from marine plants (phenols, mycosporine-like amino acids, and fucoidan) can activate the Nrf2-mediated defense pathway in keratinocytes, fibroblasts, or melanocytes challenged by UV, H_2_O_2_, TPA, TNF-α, or AAPH, thereby mitigating oxidative and inflammatory reactions. The evidence presented for the activation of the Nrf2-mediated defense pathway by natural products includes the increased nuclear translocation and/or accumulation of Nrf2, the increased ARE promoter activity, and the increased expression of ARE-driven target enzymes, such as HO-1, NQO-1, γ-GCLM, γ-GCLC, GPX, SOD, catalase, GST, UGT1A1, and OGG-1. As the mechanism of Nrf2 activation, the modification of the reactive Cys residues of Keap1 was mainly proposed, but the epigenetic regulation of *Nrf2* gene expression was also suggested [92,100]. A relatively large number of substances were tested in animal models including *Nrf2(-/-)* knockout mice [97] and human skin [108]. These “natural Nrf2 modulators” cannot necessarily function only through Nrf2-dependent mechanisms because many other bioactivities of these compounds have been previously reported [137,138,161,162].

While this review focuses on skin protection by natural Nrf2 modulators, such compounds can have similar cell protective effects in other organs. A variety of natural and synthetic Nrf2 modulators have been extensively studied for application to autoimmune diseases, chronic respiratory diseases, digestive diseases, cardiovascular diseases, metabolic diseases, neurodegenerative diseases, and cancer [137,138,163,164].

## 5. Conclusions

In conclusion, even after decades have passed since the discovery of the Nrf2-mediated defense system, this research field is growing. In the meantime, the importance of Nrf2 as the main regulator of the biological defense mechanism has been solidified. In addition, research to protect the skin by using natural products derived from terrestrial or marine plants and to apply it to the prevention and treatment of skin diseases has rapidly increased over the past decade. Therefore, using the information and technology accumulated so far, the possibility of developing a new drug using a more selective, powerful, and target specific Nrf2 modulator is increasing. It is hoped that this review will increase the understanding of the Nrf2-mediated defense mechanism for researchers entering this field and serve as a reference for designing and conducting their challenging research.

## Abbreviations

AAPH	2,2′-Azobis (2-amidinopropane) dihydrochloride
AhR	Aryl hydrocarbon receptor
AMP	Adenosine monophosphate
AMPK	AMP-activated protein kinase
AP-1	Activator protein-1
ARE	Antioxidant-responsive elements
Bach1	BTB domain and CNC homolog 1
BRG1	Brahma-related gene 1
BTB	Broad-Complex, Tramtrack and Bric-a-Brac
b-zip	Basic region-leucine zipper
cAMP	Cyclic AMP
CBP	CREB binding protein
cDNA	complementary DNA
CHD6	Chromo-ATPase/helicase DNA binding protein 6
CK2	Casein kinase 2
CNC	Cap ‘N’ Collar
COX-2	Cyclooxygenase-2
CREB	Cyclic AMP-responsive element binding protein
Crm1	Chromosome region maintenance 1
Cul	Cullin
DCNB	2,4-dinitrochlorobenzene
DGR	Double glycine repeats
DMBA	7,12-Dimethylbenz(a)anthracene
DNA	Deoxyribonucleic acid
DNMT	DNA methyltransferases
EBS	Epidermolysis bullosa simplex
ECH	Erythroid cell-derived protein with CNC homology
EpRE	Electrophile-responsive elements
ER	Endoplasmic reticulum
ERK	Extracellular signal-regulated kinase
FPN1	Ferroportin 1
γ-GCLC	γ-Glutamate-cysteine ligase catalytic subunit
γ-GCLM	γ-Glutamate-cysteine ligase regulatory subunit
GPX	Glutathione peroxidase
GR	Glucocorticoid receptor
GSK3β	Glycogen synthase kinase 3β
GST	Glutathione S-transferase
HaCaT	Human adult skin keratinocytes propagated under low Ca^2+^ conditions and elevated temperature
HDAC	Histone deacetylases
HMGB-1	High-mobility group box-1
HMG-CoA	3-Hydroxy-3-methylglutaryl-coenzyme A
HO-1	Heme oxygenase-1
HRD1	HMG-CoA reductase degradation 1
HSE	Heat-shock response transcription elements
HSP70	Heat shock protein 70
IL	Interleukin
iNOS	Inducible nitric oxide synthase
IVR	Intervening region
JNK	c-Jun N-terminal kinase
Keap1	Kelch-like ECH-associated protein 1
Lys	Lysine
MAD	Mothers against decapentaplegic
MAPK	Mitogen-activated protein kinase
MCP1	Monocyte chemoattractant protein 1
MEF	Mouse embryo fibroblasts
miR	Micro RNA
MMP	Matrix metalloproteinases
MPO	Myeloperoxidase
mRNA	Messenger RNA
MT	Metallothionein
NAD(P)H	Nicotinamide adenine dinucleotide (phosphate)
NDGA	Nordihydroguaiaretic acid
Neh	Nrf2-ECH homology
NES	Nuclear export signal
NF-E2	Nuclear factor erythroid 2
NF-κB	Nuclear factor kappa-light-chain-enhancer of activated B cells
NK	Natural killer
NLS	Nuclear localization signal
NO	Nitric oxide
Nrf2	Nuclear factor erythroid 2-related factor 2
NQO-1	NAD(P)H quinone oxidoreductase-1
NTR	N-terminal region
OGG1	8-Oxoguanine DNA glycosylase 1
PERK	PKR-like ER kinase
PG	Prostaglandin
PI3K	Phosphoinositide 3-kinase
PKB	Protein kinase B (Akt)
PKC	Protein kinase C
PKR	Protein kinase RNA-activated
RBX1	RING-box 1
RING	Really interesting new gene
RNA	Ribonucleic acid
ROS	Reactive oxygen species
RXRα	Retinoic X receptor α
sMaf	Small musculoaponeurotic fibrosarcoma
SA-β-Gal	Senescence associated-β-galactosidase
SAMP1	Senescence-accelerated mouse prone 1
SCID	Severe combined immunodeficiency
Ser	Serine
Sirt	Sirtuin (silent mating type information regulation 2 homolog)
Skp1	S-Phase kinase associated protein 1
Smad3	Similarity to the Drosophila gene MAD 3
SOD	Superoxide dismutase
STAT1	Signal transduction and activation of transcription 1
β-TrCP	β-Transducin repeat-containing protein
UDP	Uridine diphosphate
UGT1A1	UDP-glucuronosyltransferase 1A1
TGFβ	Transforming growth factor β
TβR	TGFβ receptor
Thr	Threonine
TNF-α	Tumor necrosis factor-α
TPA	Tetradecanoylphorbol-13-acetate
Tyr	Tyrosine
